# Transient receptor potential ankyrin 1 (TRPA1) mediates reactive oxygen species-induced Ca^2+^ entry, mitochondrial dysfunction, and caspase-3/7 activation in primary cultures of metastatic colorectal carcinoma cells

**DOI:** 10.1038/s41420-023-01530-x

**Published:** 2023-07-01

**Authors:** Pawan Faris, Agnese Rumolo, Giorgia Pellavio, Matteo Tanzi, Mauro Vismara, Roberto Berra-Romani, Andrea Gerbino, Salvatore Corallo, Paolo Pedrazzoli, Umberto Laforenza, Daniela Montagna, Francesco Moccia

**Affiliations:** 1 Department of Biology and Biotechnology “Lazzaro Spallanzani”, University of Pavia, via Forlanini 6, 27100 Pavia, Italy; 2 Foundation IRCCS Policlinico San Matteo, Laboratory of Immunology Transplantation, Piazzale Golgi 19, Pavia, Italy; 3 Department of Molecular Medicine, University of Pavia, via Forlanini 6, 27100 Pavia, Italy; 4 Department of Biomedicine, School of Medicine, Benemérita Universidad Autónoma de Puebla, 13 Sur 2702 Colonia Volcanes, Puebla, 72410 Mexico; 5 Department of Biosciences, Biotechnologies and Environment, University of Bari Aldo Moro, Via G. Amendola 165/A, 70125 Bari, Italy; 6 Medical Oncology, Foundation IRCCS Policlinico San Matteo, Piazzale Golgi 19, 27100 Pavia, Italy; 7 Department of Sciences Clinic-Surgical, Diagnostic and Pediatric, University of Pavia, Pavia, Italy

**Keywords:** Cancer, Physiology

## Abstract

Colorectal carcinoma (CRC) represents the fourth most common cancer worldwide and is the third most common cause of malignancy-associated mortality. Distant metastases to the liver and lungs are the main drivers of CRC-dependent death. Pro-oxidant therapies, which halt disease progression by exacerbating oxidative stress, represent an antitumour strategy that is currently exploited by chemotherapy and ionizing radiation. A more selective strategy to therapeutically exploit reactive oxygen species (ROS) signaling would consist in targeting a redox sensor that is up-regulated in metastatic cells and is tightly coupled to the stimulation of cancer cell death programs. The non-selective cation channel, Transient Receptor Potential Ankyrin 1 (TRPA1), serves as a sensor of the cellular redox state, being activated to promote extracellular Ca^2+^ entry by an increase in oxidative stress. Recent work demonstrated that TRPA1 channel protein is up-regulated in several cancer types and that TRPA1-mediated Ca^2+^ signals can either engage an antiapoptotic pro-survival signaling pathway or to promote mitochondrial Ca^2+^ dysfunction and apoptosis. Herein, we sought to assess for the first time the outcome of TRPA1 activation by ROS on primary cultures of metastatic colorectal carcinoma (mCRC cells). We found that TRPA1 channel protein is up-regulated and mediates enhanced hydrogen peroxide (H_2_O_2_)-induced Ca^2+^ entry in mCRC cells as compared to non-neoplastic control cells. The lipid peroxidation product 4-hydroxynonenal (4-HNE) is the main ROS responsible for TRPA1 activation upon mCRC cell exposure to oxidative stress. TRPA1-mediated Ca^2+^ entry in response to H_2_O_2_ and 4-HNE results in mitochondrial Ca^2+^ overload, followed by mitochondrial depolarization and caspase-3/7 activation. Therefore, targeting TRPA1 could represent an alternative strategy to eradicate metastatic CRC by enhancing its sensitivity to oxidative stress.

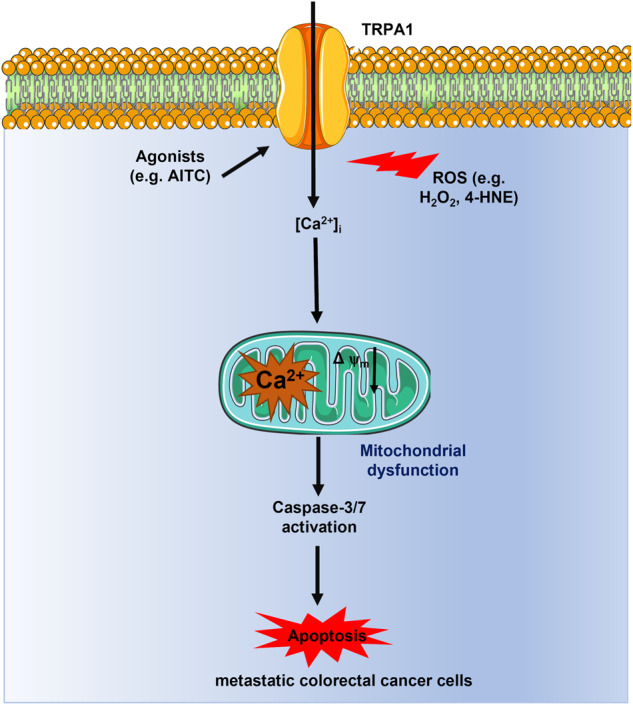

## Introduction

Colorectal carcinoma (CRC) represents the fourth most common cancer worldwide and is the third most common cause of malignancy-associated mortality, being responsible for 9.2% of fatalities among oncological patients (International Agency for Research on Cancer Available from: https://gco.iarc.fr/today, accessed 4^th^/04/2023). Involvement of secondary organs, such as the liver and lungs, is the main driver of CRC-dependent death: 25% of CRC patients show metastatic disease at diagnosis, whereas ≈50% of the patients develop disease recurrence within 5 years from surgery or adjuvant treatment [1]. The development of more effective strategies after the failure of conventional therapies for advanced/recurrent disease represents an unmet need for CRC patients. Reactive oxygen species (ROS), such as hydrogen peroxide (H_2_O_2_), have long been known to fuel tumor metastasis and invasion in a variety of cancer types [2], including mCRC [3]. Nevertheless, human clinical trials showed that dietary supplementation with antioxidants did not decrease, but rather enhanced, cancer incidence and cancer-related mortality [4]. Pro-oxidant therapies [2, 4], which halt disease progression by exacerbating oxidative stress in cancer cells, may represent an effective alternative antitumour strategy to current systemic treatments that are associated with a number of harmful side effects often leading to impaired quality of life, a worse overall prognosis and waste of health care resources [5]. A more selective strategy to exploit ROS signaling for therapeutic purposes would consist in targeting a redox sensor that is up-regulated in neoplastic cells and is tightly coupled to the stimulation of cancer cell death programs.

Transient receptor potential ankyrin 1 (TRPA1) is a non-selective cation channel that is located within the plasma membrane and promotes extracellular Ca^2+^ entry in response to multiple chemical, physical, and thermal stimuli, thereby serving a polymodal sensor [6]. TRPA1 may serve as a ROS sensor due to the abundance of hyper-reactive cysteine residues that are located at the NH_2_-terminal and can be oxidized by H_2_O_2_ [6, 7]. TRPA1 is the most abundant redox-sensitive TRP isoform in most cancer types [8], including invasive ductal breast carcinoma and lung adenocarcinoma, in which it supports H_2_O_2_-evoked intracellular Ca^2+^ oscillations and Ca^2+^-dependent recruitment of pro-survival and antiapoptotic pathways to prevent ROS-induced cancer cell death [9]. Conversely, TRPA1-mediated increase in intracellular Ca^2+^ concentration ([Ca^2+^]_i_) supports H_2_O_2_-induced mitochondrial damage and apoptosis in other types of solid malignancies, such as glioblastoma multiforme [10, 11] and human oral squamous cell carcinoma (OSCC) [12]. A series of recent studies demonstrated that intracellular Ca^2+^ signals may either stimulate proliferation [13], inhibit the cell-cycle [14], or induce cell death [15] in primary cultures of metastatic CRC (mCRC) cells. The Ca^2+^ source dictates the outcome of [Ca^2+^]_i_ rise on cell fate, as distinct Ca^2+^-permeable channels can be selectively coupled to different Ca^2+^-dependent decoders in cancer cells [16, 17]. A further layer of complexity to the Ca^2+^-dependent regulation of cancer hallmarks is added by the evidence that the same TRP isoform, e.g., TRP Vanilloid 1 (TRPV1), can exert opposing effects in different cancer types [18]. Therefore, understanding whether TRPA1-mediated Ca^2+^ entry stimulates or rather prevents ROS-dependent mCRC dell death is mandatory to design alternative therapies based upon the manipulation of TRPA1 activity to sensitize mCRC cells to oxidative stress.

## Results

### TRPA1 protein is up-regulated and mediates enhanced Ca^2+^ entry in mCRC cells

**Fig. 1:**
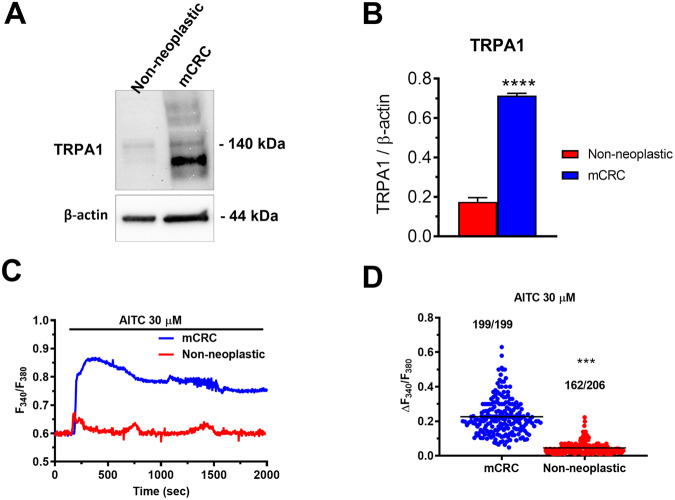
TRPA1 protein is up-regulated and mediates enhanced Ca^2+^ signaling in primary cultures of mCRC cells. **A** TRPA1 protein expression in non-neoplastic cells and primary cultures of mCRC cells. Blots representative of four independent experiments (each conducted on samples deriving from a distinct patient) were shown. Major bands of the predicted molecular weights for TRPA1 and β-actin proteins were indicated. **B** Mean ± SE of TRPA1 protein expression in non-neoplastic and mCRC cells. The results were normalized to the corresponding β-actin (****p < 0.0001; Student’s *t*-test). TRPA1 protein was significantly more expressed in mCRC cells. **C** The selective TRPA1 agonist, AITC (30 µM), evoked intracellular Ca^2+^ signals in mCRC and non-neoplastic cells. **D** Mean ± SE of the amplitude of the peak Ca^2+^ response (scattered dot plot) to AITC in both mCRC and non-neoplastic cells. Student’s *t*-test: ***p < 0.001. The numbers placed above the scattered dots represent the number of responding cells out of the total cell number. *N* = 4 for each experimental condition.

**Fig. 2:**
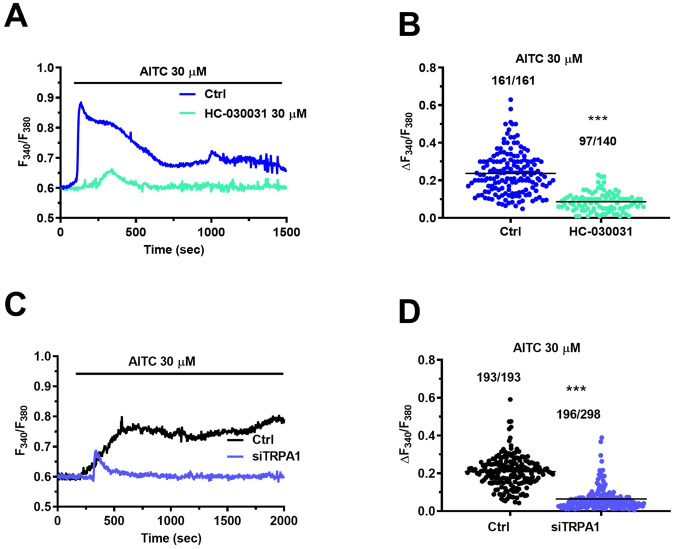
TRPA1 channel mediates AITC-evoked Ca^2+^ entry in primary cultures of mCRC cells. **A** Intracellular Ca^2+^ signals induced by 30 µM AITC (Ctrl) were abrogated upon pre-treating mCRC cells with the specific TRPA1 inhibitor, HC-030031 (30 µM, 30 min). **B** Mean ± SE of the peak Ca^2+^ signal evoked by AITC in the absence (Ctrl) and presence of HC-030031. **C** Intracellular Ca^2+^ signals induced by 30 µM AITC in mCRC cells transfected with a scrambled construct (Ctrl) or with a selective siTRPA1. **D** Mean ± SE of the amplitude of Ca^2+^ response to AITC under the designated treatment. Student’s *t*-test: ***p < 0.001. The placed above the scattered dots represent the number of responding cells out of the total cell number. *N* = 4 for each experimental condition.

Preliminary evidence indicates that *TRPA1* gene is expressed in CRC [19], but it is still unknown whether it is translated into a functional protein in mCRC cells. Immunoblots identified a major band of ∼140 kDa in both primary cultures of mCRC and control cells isolated from adjacent non-neoplastic tissue (Fig. 1A and Fig. S1), as also observed in other cancer cell types [20, 21], and densitometric analysis revealed that TRPA1 protein was significantly (*p* < 0.05) up-regulated in mCRC cells (Fig. 1B). In order to assess whether TRPA1 protein was able to mediate extracellular Ca^2+^ entry, both cell types were loaded with the Ca^2+^-sensitive fluorophore, Fura-2 acetoxymethyl ester (Fura-2/AM), as described elsewhere [14, 22]. TRPA1 stimulation by the selective electrophilic agonist, allyl isothiocyanate (AITC; 30 µM) induces larger intracellular Ca^2+^ signals in primary cultures of mCRC cells as compared to non-neoplastic cells (Fig. 1C, D). Interestingly, AITC induced a sustained Ca^2+^ overload in mCRC cells (Fig. 1A, blue tracing), which is a hallmark of pro-apoptotic Ca^2+^ signals [11, 23], while it evoked low-amplitude intracellular Ca^2+^ oscillations in non-neoplastic cells (Fig. 1C; red tracing), which could rather exert a mitogenic effect [13, 24]. AITC failed to increase [Ca^2+^]_i_ in the absence of extracellular Ca^2+^ (0Ca^2+^) (Fig. S2), while restoring extracellular Ca^2+^ concentration (1.5 mM) caused an immediate and long-lasting elevation in [Ca^2+^]_i_ in mCRC cells (Fig. S2). Therefore, the Ca^2+^ response to AITC is mainly mediated by extracellular Ca^2+^ entry. In order to confirm that TRPA1 mediates AITC-evoked Ca^2+^ influx, mCRC cells were pretreated with HC-030031 (30 µM), which represents the most widespread used TRPA1 inhibitor [6, 7, 21, 23]. As expected, HC-030031 significantly (*p* < 0.05) reduced both the amplitude and the duration of the Ca^2+^ response to AITC (Fig. 2A, B). In addition, genetic silencing of TRPA1 expression with a selective small interfering RNA (siTRPA1) significantly (*p* < 0.05) reduced AITC-evoked extracellular Ca^2+^ entry in mCRC cells (Fig. 2C, D). The efficacy of TRPA1 deletion in mCRC cells by the siTRPA1 was confirmed by comparing TRPA1 protein expression in mCRC cells transfected with the selective siTRPA1 and with a scrambled construct (Figs. S3 and S4). Altogether, these data show that TRPA1 protein is up-regulated and mediated extracellular Ca^2+^ entry in primary cultures of mCRC cells.

### TRPA1 mediates H_2_O_2_-induced Ca^2+^ signals in mCRC cells

**Fig. 3:**
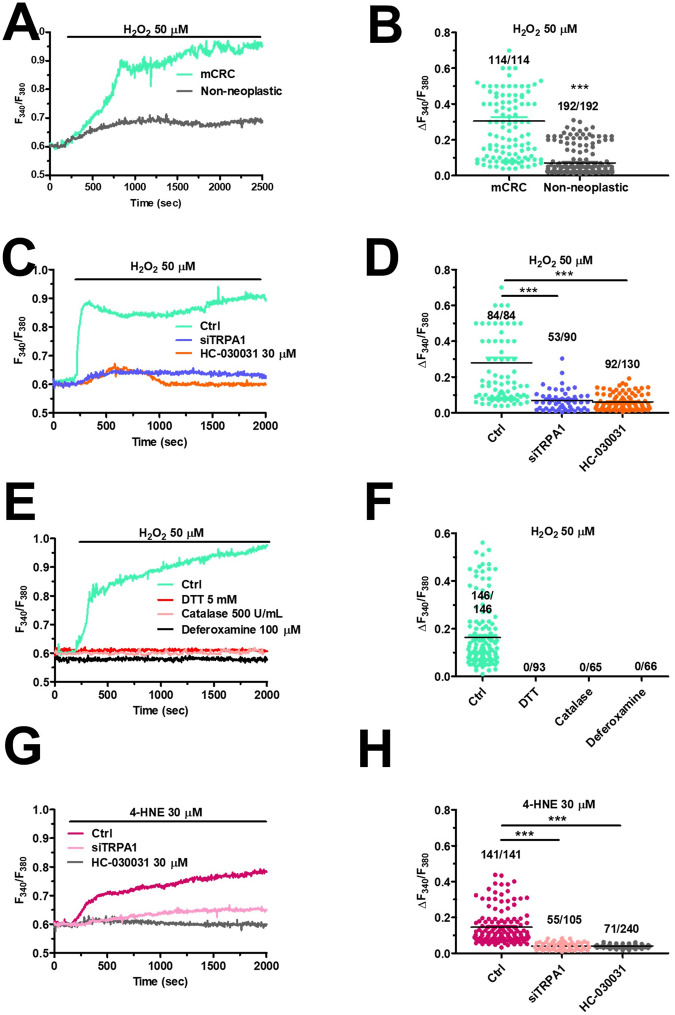
TRPA1 mediates H_2_O_2_-induced intracellular Ca^2+^ signals in primary cultures of mCRC cells. **A** H_2_O_2_ (50 µM) induces larger Ca^2+^ signals in mCRC as compared to non-neoplastic cells. **B** Mean ± SE of peak Ca^2+^ signal evoked by H_2_O_2_ in both mCRC and non-neoplastic cells. Student’s *t*-test: ***p < 0.001. The placed above the scattered dots represent the number of responding cells out of the total cell number. *N* = 4 for each experimental condition. **C** Intracellular Ca^2+^ signals induced by 50 µM H_2_O_2_ (Ctrl) were abrogated in mCRC cells pretreated with the specific TRPA1 inhibitor, HC-030031 (30 µM, 30 min), or transfected with the selective siTRPA1. **D** Mean ± SE of the peak Ca^2+^ signal evoked by H_2_O_2_ in control (Ctrl) mCRC cells and in mCRC cells transfected with siTRPA1 or pretreated with HC-030031. One-way ANOVA followed by the post hoc Dunnett’s test: ***p < 0.001. The numbers placed above the scattered dots represent the number of responding cells out of the total cell number. *N* = 4 for each experimental condition. **E** Intracellular Ca^2+^ signals induced by 50 µM H_2_O_2_ in the absence (Ctrl) and presence of the thiol-reducing compound, DTT (5 µM), the H_2_O_2_ scavenger, catalase (500 U/mL) or the iron-chelating compound, deferoxamine (100 µM). **F** Mean ± SE of peak Ca^2+^ signal evoked by H_2_O_2_ under the designated treatments. The numbers placed above the scattered dots represent the number of responding cells out of the total cell number. *N* = 4 for each experimental condition. **G** Intracellular Ca^2+^ signals induced by the selective TRPA1 agonist, 4-HNE (30 µM), in mCRC cells under control conditions (Ctrl) or upon pharmacological (HC-030031; 30 µM, 30 min) or genetic (with a selective siTRPA1) blockade of TRPA1 activity. **H** Mean ± SE of peak Ca^2+^ signal evoked by 4-HNE in control (Ctrl) mCRC cells and in mCRC cells treated with DTT, catalase, and deferoxamine. One-way ANOVA followed by the post hoc Dunnett’s test: ***p < 0.001. The numbers placed above the scattered dots represent the number of responding cells out of the total cell number. *N* = 4 for each experimental condition.

In cancer cells, H_2_O_2_ may either stimulate ROS-dependent apoptosis [11] or engage an anti-oxidant defense program through intracellular Ca^2+^ signaling. Preliminary Ca^2+^ imaging recordings showed that H_2_O_2_ induced a dose-dependent increase in [Ca^2+^]_i_ (Fig. S5A), which presented a minimum effective dose of 1 µM, a half-maximal effective concentration (EC_50_) of 35.37 µM, and a maximal response at 200 µM (Fig. S5B). Low micromolar doses of H_2_O_2_ (10–25 µM) induced low-amplitude intracellular Ca^2+^ oscillations (Fig. S5A), while higher doses evoked a potentially cytotoxic Ca^2+^ overload (Figure S5A), as previously described for AITC (Fig. 1C, blue tracing). H_2_O_2_ concentration within cancer microenvironment may rise to 50 µM [25]. The Ca^2+^ response to 50 µM H_2_O_2_ was significantly (*p* < 0.05) larger in mCRC cells as compared to non-neoplastic cells (Fig. 3A, B). Furthermore, the prolonged increase in [Ca^2+^]_i_ evoked by 50 µM H_2_O_2_ in mCRC cells was dampened by pharmacological (via 30 µM HC-030031) and genetic (via the selective siTRPA1) blockade of TRPA1 (Fig. 3C and Fig. 3D). In addition, the Ca^2+^ response to was sensitive to dithiothreitol (DTT) (5 mM) (Fig. 3E, F), a thiol-reducing compound that reverses H_2_O_2_-dependent Ca^2+^ signals [26, 27], and to the H_2_O_2_ scavenger, catalase (500 U/mL) (Fig. 3E, F) [26, 28]. Oxidative stress in cancer microenvironment may result in the peroxidation of ω6 polyunsaturated fatty acids in the plasma membrane, thereby leading to the formation of 4-hydroxy-nonenal (4-HNE) [9, 29]. 4-HNE has recently been shown to stimulate TRPA1-mediated Ca^2+^ influx in several cell types [30, 31], including melanoma cell lines [29]. Fifty µM H_2_O_2_-evoked Ca^2+^ overload in mCRC cells was abolished by deferoxamine (100 µM) (Fig. 3E, F), which prevents H_2_O_2_ degradation into the hydroxyl radical (OH^•^) [26, 31]. Furthermore, exogenous administration of 4-HNE (30 µM) induced a slowly rising and protracted increase in [Ca^2+^]_i_ that was sensitive to TRPA1 inhibition with HC-030031 (30 µM) (Fig. 3G, H). Therefore, these data demonstrated that high concentrations of H_2_O_2_ induced cytosolic Ca^2+^ overload via 4-HNE-dependent TRPA1 activation in mCRC cells.

### TRPA1 mediates H_2_O_2_-induced mitochondrial dysfunction and caspase-3/7 activation in mCRC cells

In order to assess whether H_2_O_2_-induced TRPA1 activation affect mCRC cell viability, we exploited the Trypan blue exclusion assay [14]. AITC (30 µM) and H_2_O_2_ (50 µM) caused a significant (*p* < 0.05) reduction in the percentage of viable cells at 24 h, 48 h, and 72 h (Fig. S6A, C, respectively). However, the pharmacological blockade of TRPA1 with HC-030031 (30 µM) rescued viability in mCRC cells exposed both to AITC (Fig. S6A) and H_2_O_2_ (Fig. S6C). The reduction in cell viability was associated to a significant (*p* < 0.05) decrease in cell growth that was rescued by blocking TRPA1-mediated Ca^2+^ influx with HC-030031 (30 µM) (Fig. S6B, S6D for AITC and H_2_O_2_, respectively). Conversely, stimulating TRPA1 with either AITC (30 µM) or H_2_O_2_ (50 µM) did not affect viability (Fig. S6E) and cell growth (Fig. S6F) in non-neoplastic cells. Therefore, these preliminary findings indicate that TRPA1-mediated Ca^2+^ influx affects viability in primary cultures of mCRC cells, but not in their normal counterparts.

**Fig. 4:**
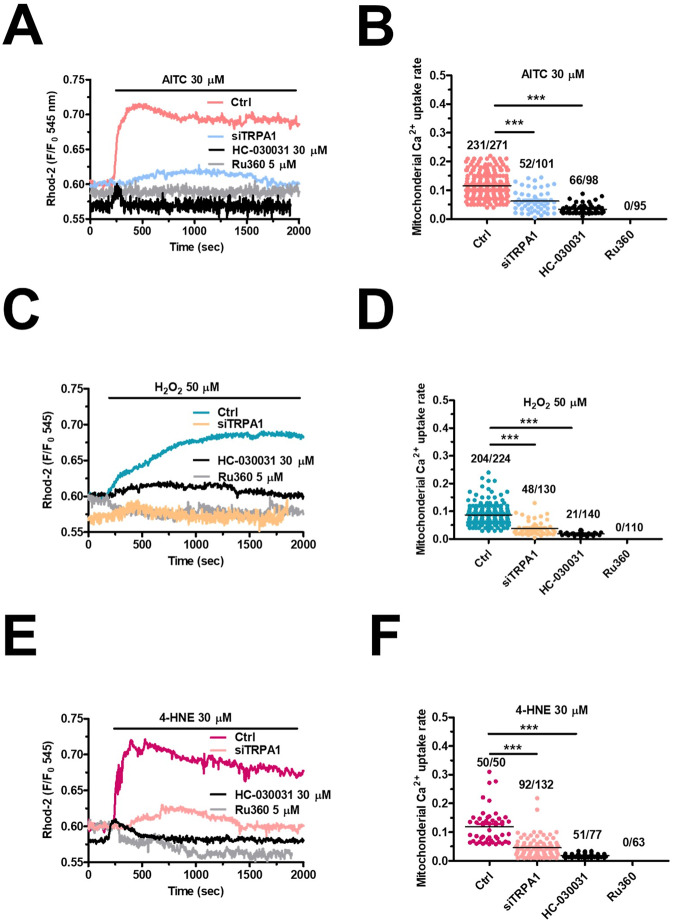
TRPA1 activation induces mitochondrial Ca^2+^ overload in primary cultures of mCRC cells. **A** Mitochondrial Ca^2+^ signals induced by AITC (30 µM) in mCRC cells maintained under control conditions (Ctrl), pretreated with HC-030031 (30 µM, 30 min) or with the highly specific inhibitor of the mitochondrial Ca^2+^ uniporter, Ru360 (5 µM, 30 min), or transfected with the selective siTRPA1. **B** Mean ± SE of peak mitochondrial Ca^2+^ signal evoked by AITC in control (Ctrl) mCRC cells and in mCRC cells transfected with siTRPA1 or pretreated with HC-030031 or Ru360. One-way ANOVA followed by the post hoc Dunnett’s test. ***p < 0.001. The numbers placed above the scattered dots represent the number of responding cells out of the total cell number. *N* = 4 for each experimental condition. **C** Mitochondrial Ca^2+^ signals induced by H_2_O_2_ (50 µM) in mCRC cells maintained under control conditions (Ctrl), pretreated with HC-030031 (30 µM, 30 min) or with the highly specific inhibitor of the mitochondrial Ca^2+^ uniporter, Ru360 (5 µM, 30 min), or transfected with the selective siTRPA1. **D** Mean ± SE of peak mitochondrial Ca^2+^ signal evoked by H_2_O_2_ in control (Ctrl) mCRC cells and in mCRC cells transfected with siTRPA1 or pretreated with HC-030031 or Ru360. One-way ANOVA followed by the post hoc Dunnett’s test. ***p < 0.001. The numbers placed above the scattered dots represent the number of responding cells out of the total cell number. *N* = 4 for each experimental condition. **E** Mitochondrial Ca^2+^ signals induced by 4-HNE (30 µM) in mCRC cells maintained under control conditions (Ctrl), pretreated with HC-030031 (30 µM, 30 min) or with the highly specific inhibitor of the mitochondrial Ca^2+^ uniporter, Ru360 (5 µM, 30 min), or transfected with the selective siTRPA1. **F** Mean ± SE of peak mitochondrial Ca^2+^ signal evoked by 4-HNE in control (Ctrl) mCRC cells and in mCRC cells transfected with siTRPA1 or pretreated with HC-030031 or Ru360. One-way ANOVA followed by the post hoc Dunnett’s test. ***p < 0.001. The numbers placed above the scattered dots represent the number of responding cells out of the total cell number. *N* = 4 for each experimental condition.

**Fig. 5:**
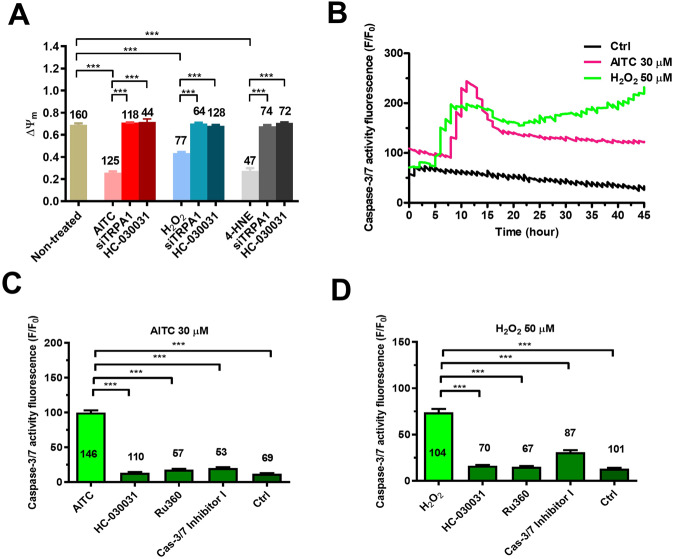
TRPA1-mediated extracellular Ca^2+^ entry causes mitochondrial depolarization and caspase-3/7 activation in mCRC cells. **A** Mean ± SE of ∆Ψ_m_ measured under control conditions (Ctrl) and after the following treatments: (1) AITC (30 µM, 6 h); AITC (30 µM, 6 h) + HC-030031 (30 µM, 30 min); AITC (30 µM, 6 h) + siTRPA1; (2) H_2_O_2_ (50 µM, 6 h); H_2_O_2_ (50 µM, 6 h) + HC-030031 (30 µM, 30 min); H_2_O_2_ (50 µM, 6 h) + siTRPA1; (3) 4-HNE (30 µM, 6 h); 4-HNE (30 µM, 6 h) + HC-030031 (30 µM, 30 min); 4-HNE (30 µM, 6 h) + siTRPA1. One-way ANOVA followed by the post hoc Dunnett’s test. ***p < 0.001. The numbers placed above the histogram bars represent the number of responding cells out of the total cell number. *N* = 4 for each experimental condition. ∆Ψ_m_ was measured by evaluating tetramethyl rhodamine methyl ester (TMRM) fluorescence. **B** Tracings show the changes in CellEvent^TM^ fluorescence, signifying caspase-3/7 activation, in the absence (Ctrl) and presence of either AITC (30 µM) or H_2_O_2_ (50 µM). Each tracing is representative of 113 cells (Ctrl), 91 cells (AITC), and 86 cells (H_2_O_2_) from 3 independent experiments. Every recording lasted 45 h at a sampling rate of 1 image/15 min. **C** Mean ± SE of CellEvent^TM^ fluorescence intensity under the following conditions: Control (Ctrl); AITC (30 µM, 6 h); AITC (30 µM, 6 h) + HC-030031 (30 µM, 30 min); AITC (30 µM, 6 h) + Ru360 (5 µM, 30 min); AITC (30 µM, 6 h) + Caspase-3/7 Inhibitor I (20 µM, 30 min). One-way ANOVA followed by the post hoc Dunnett’s test. ***p < 0.001. The numbers placed above the histogram bars represent the number of responding cells out of the total cell number. *N* = 4 for each experimental condition. **D** Mean ± SE of CellEvent^TM^ fluorescence intensity under the following conditions: Control (Ctrl); H_2_O_2_ (50 µM, 6 h); H_2_O_2_ (50 µM, 6 h) + HC-030031 (30 µM, 30 min); H_2_O_2_ (50 µM, 6 h) + Ru360 (5 µM, 30 min); H_2_O_2_ (50 µM, 6 h) + Caspase-3/7 Inhibitor I (20 µM, 30 min). One-way ANOVA followed by the post hoc Dunnett’s test. ***p < 0.001. The numbers *p*laced above the histogram bars represent the number of responding cells out of the total cell number. *N* = 4 for each experimental condition.

We then evaluated whether TRPA1 activation in mCRC cells leads to apoptosis. A hallmark of apoptotic cell death is represented by mitochondrial Ca^2+^ overload, which causes mitochondrial depolarization and opening of the mitochondrial permeability transition pore (mPTP) followed by caspase-3/7 activation [32–34]. The ROS-dependent increase in mitochondrial Ca^2+^ concentration ([Ca^2+^]_mito_) was evaluated in mCRC cells loaded with Rhod-2/AM, the most widely employed fluorophore to monitor mitochondrial free Ca^2+^ levels [32, 34, 35]. AITC (30 µM) evoked a long-lasting elevation in [Ca^2+^]_mito_ that was abolished by blocking TRPA1 with either HC-030031 (30 µM) or the selective siTRPA1 and by inhibiting mitochondrial Ca^2+^ uptake with the specific antagonist Ru360 (5 µM) [35] (Fig. 4A, B). Also, 50 µM H_2_O_2_ (Fig. 4C, D) and 30 µM 4-HNE (Fig. 4E, 4F) induced a protracted elevation in [Ca^2+^]_mito_ that was sensitive to the genetic or pharmacological blockade of TRPA1-mediated Ca^2+^ entry and to the pharmacological blockade of mitochondrial Ca^2+^ uptake. Consistently, exposure to 30 µM AITC (Fig. 5A), 50 µM H_2_O_2_ (Fig. 5A), and 30 µM 4-HNE (Fig. 5A) caused significant (*p* < 0.05) mitochondrial depolarization, which was rescued by blocking TRPA1-mediated Ca^2+^ entry with either HC-030031 (30 µM) or the selective siTRPA1 (Fig. 5A). We finally assessed whether TRPA1-mediated mitochondrial Ca^2+^ overload results in caspase activation in mCRC cells loaded with the with the caspase-3/7-sensitive DEVD-based dye CellEvent [36, 37]. Single-cell imaging revealed that both AITC (30 µM) and H_2_O_2_ (50 µM) caused a significant increase in caspase-3/7 activation at, respectively, ∼12.5 and ∼7.5 h, which was maintained until the end of the recording period (45 h) (Fig. 5B and Fig. S7A). Caspase-3/7 activation was suppressed by blocking TRPA1 with HC-03031 (30 µM), by inhibiting MCU with Ru360 (5 µM), and by preventing caspase-3/7 activation with Caspase-3/7 Inhibitor I (20 µM) (Fig. 5C for AITC and Fig. 5D for H_2_O_2_; see also Fig. S7B for both agonists). These findings indicate that TRPA1-mediated Ca^2+^ influx supports H_2_O_2_-induced mCRC cell apoptotic death by causing mitochondria dysfunction and caspase-3/7 activation.

## Discussion

In this investigation, we demonstrated for the first time that the redox-sensitive TRPA1 channel is up-regulated, mediates enhanced Ca^2+^ entry and thereby leads to mitochondrial dysfunction and caspase-3/7 activation in primary cultures of mCRC. Therefore, TRPA1 stimulation could represent an alternative therapeutic approach to sensitize mCRC to ROS-dependent cell death [38].

TRPA1 is emerging as the primary redox-sensitive TRP isoform in cancer microenvironment [7–9]. TRPA1 protein is up-regulated in multiple solid malignancies, such as invasive ductal breast carcinoma and lung adenocarcinoma [9], OSCC [12], pancreatic adenocarcinoma [20], and prostate cancer [21]. The outcome of TRPA1 stimulation by oxidative stress may vary depending on the tumor type: for instance, TRPA1-mediated Ca^2+^ entry engages a non-canonical anti-oxidant defense program in lung and breast cancers [8, 9], while it stimulates mitochondrial dysfunction and apoptosis in glioblastoma multiforme [10, 11]. Herein, we found that TRPA1 protein expression was remarkably enhanced in primary cultures of mCRC cells, which represent a suitable model to investigate the impact of intracellular Ca^2+^ signals on a therapeutically relevant model of human CRC [13–15, 39, 40], as compared to non-neoplastic cells. In addition, the electrophilic TRPA1 agonist, AITC, evoked a sustained increase in [Ca^2+^]_i_ that was sensitive to both pharmacological (via HC-030031) and genetic blockade of TRPA1 activity (via a selective siTRPA1). The waveform of this Ca^2+^ response is quite different from the repetitive oscillations in [Ca^2+^]_i_ evoked by TRPA1 activation in lung and breast cancer cells [9]. Interestingly, intracellular Ca^2+^ oscillations in cancer cells have long been known to stimulate cell proliferation and survival [41, 42], while long-lasting elevations in [Ca^2+^]_i_ lead to apoptotic cell death [32, 41, 43]. In accord, AITC-evoked cytosolic Ca^2+^ overload reduced viability in OSCC cells [12].

Similar to AITC, mid-to-high micromolar concentrations of H_2_O_2_ evoked a long-lasting increase in [Ca^2+^]_i_, which was inhibited by blocking TRPA1-mediated Ca^2+^ entry via either HC-030031 or the selective siTRPA1. Reactive lipid mediators generated by lipid peroxidation of polyunsaturated fatty acids in the plasma membrane, such as 4-HNE, are involved in cancer initiation and progression [9, 29, 44]. TRPA1 is highly sensitive to 4-HNE [30, 31] and 4-HNE-induced TRPA1 activation has been reported in melanoma cell lines [29]. In the presence of iron, H_2_O_2_ is degraded into OH^•^ via the Fenton reaction, thereby inducing lipid peroxidation and 4-HNE formation [45, 46]. Of note, the Ca^2+^ response to H_2_O_2_ was abolished by preventing the Fenton reaction with deferoxamine. In addition, exogenous administration of 4-HNE caused a cytosolic Ca^2+^ overload in mCRC cells that resembled those induced by AITC and H_2_O_2_ and was dependent on TRPA1 activation. These findings strongly indicate that 4-HNE is the more likely agonist to induce TRPA1 activation by oxidative stress in mCRC cells. Moreover, the long-lasting Ca^2+^ elevation resulting from TRPA1 stimulation in mCRC cells is seemingly more suitable to stimulate mitochondrial dysfunction and apoptotic cell death rather than promoting cell proliferation or survival. In accord, prolonged exposure of primary cultures of mCRC cells to AITC and H_2_O_2_ reduced their viability and proliferation rate in a TRPA1-dependent manner. Furthermore, stimulation with AITC, H_2_O_2_, and 4-HNE caused mitochondrial Ca^2+^ overload that was suppressed by the pharmacological and genetic blockade of TRPA1-mediated extracellular Ca^2+^ entry. This is the first evidence that TRPA1 activation leads to mitochondrial Ca^2+^ uptake in cancer cells. However, previous studies showed that TRPA1 mediates ROS-induced mitochondrial Ca^2+^ entry in OLN-93 oligodendrocytes [47] and THP-1-derived macrophages [48]. In both cell types, TRPA1-dependent mitochondrial Ca^2+^ overload led to mitochondrial depolarization and apoptotic cell death [47, 48]. In accord, aberrant mitochondrial Ca^2+^ rise induces mPTP opening and thereby leads to the dissipation of the mitochondrial membrane potential and the release of pro-apoptotic factors that activate the executioner caspase-3 and caspase-7 [33, 49, 50]. Similarly, we first found that stimulation of TRPA1 with AITC, H_2_O_2_, and 4-HNE caused a significant reduction in mitochondrial membrane potential in mCRC cells. Then, by using the commercial kit CellEvent™ Caspase-3/7 Green Detection Reagent [36, 37], we demonstrated that AITC and H_2_O_2_ evoked an early increase in caspase-3/7 activation, which was suppressed by inhibiting TRPA1-mediated Ca^2+^ entry. Likewise, TRPA1 was found to mediate oxidative stress-dependent caspase-3 activation and apoptosis in temozolomide-treated SH-SY5Y neuroblastoma cells [51] and in mouse retina undergoing ischemia-reperfusion injury [52]. Therefore, TRPA1 activation in mCRC cells supports ROS-dependent apoptosis rather than cell survival, as otherwise reported in breast and lung cancers [8, 9].

The distinct outcome of ROS-dependent TRPA1 activation in different cancer types, e.g., survival in breast and lung cancers [8, 9] and apoptosis in mCRC and glioblastoma multiforme [11], is likely to be associated to the heterogeneity of TRPA1-mediated Ca^2+^ signals. Takahashi and coworkers reported that H_2_O_2_ evoked intracellular Ca^2+^ oscillations in several breast and lung cancer cell lines. These repetitive Ca^2+^ transients in turn recruit the Ca^2+^/Calmodulin-dependent protein tyrosine kinase 2 (PYK2), which engages the anti-oxidant and antiapoptotic signaling pathways that protect cancer cells from oxidative stress [8, 9]. Of note, repetitive oscillations in [Ca^2+^]_i_ are nicely suited to recruit Ca^2+^-dependent effectors that promote cancer cell proliferation and survival, including Pyk2 [24, 53, 54], while avoiding mitochondrial Ca^2+^ overload [55]. It is still to understand why TRPA1-mediated Ca^2+^ entry does not result in repetitive Ca^2+^ spikes also in mCRC cells. The spiking Ca^2+^ response observed in breast and cancer cell lines resembles the inositol-1,4,5-trisphosphate-evoked Ca^2+^ release events from the endoplasmic reticulum (ER) that could be triggered by Ca^2+^ entry through TRP channels via the Ca^2+^-induced Ca^2+^ release process [26, 56]. Future work will have to examine the possibility that TRPA1 channels on the plasma membrane are juxtaposed to ER-located inositol-1,4,5-trisphosphate receptors in some, e.g., lung and breast cancers, but not all solid malignancies.

## Conclusions

Our results show that the redox-sensitive TRPA1 channel is up-regulated and mediates enhanced extracellular Ca^2+^ entry in mCRC cells as compared to non-neoplastic controls. The enhanced expression of TRPA1 results in cytosolic Ca^2+^ overload in mCRC cells exposed to H_2_O_2_ and this influx of Ca^2+^ is likely to depend on H_2_O_2_ degradation to OH^•^ and subsequent formation of the lipid peroxidation-derived 4-HNE. ROS-dependent TRPA1 activation in turn causes mitochondrial Ca^2+^ overload and thereby leads to mitochondrial depolarization and caspase-3/7 activation. Therefore, TRPA1 activation contributes to ROS-dependent mCRC apoptosis. These findings suggest that TRPA1 stimulation could represent a promising therapeutic avenue to sensitize mCRC cell to oxidative stress, possibly in combination with pro-oxidant therapies [2, 4].

## Materials and methods

### Isolation and expansion of mCRC cells from CRC patients

Primary mCRC cells were isolated and expanded how illustrated in [15, 39, 40]. Patients (>18 years) suffering mCRC, previously undergoing surgery intervention to excise primary CRC tumor and/or liver metastases, signed an informed consent before being enrolled. The whole procedure was carried out in according with the rules of the revised (2013) Declaration of Helsinki of 1975 (https://www.wma.net/what-we-do/medical-ethics/declaration-of-helsinki/). The Foundation IRCCS Policlinico San Matteo in Pavia (Italy) (Ethical code 20110000996, 17/01/2011) approved the present investigation. Tumor specimens were treated by using in combination the Tumor dissociation Kit (Miltenyi Biotec, Bologna, Italy; cat# 130-095-929) and the GentleMACS Dissociator (Miltenyi Biotec, Bologna, Italy, cat# 130-093-235) to rapidly generated single-cell suspensions [15, 39, 40]. Subsequently, clusters of mCRC cells were removed through filtration, and the cells were resuspended at a concentration of 0.5–1 × 10^**6**^ cells/mL in CellGro SCGM medium (Cell Genix, Freiburg, Germany, cat# 20802-0500), which was supplemented with 0.1% gentamycin (Gibco, Life Technologies Limited, Paisley, UK, cat# 15750-037) and 20% foetal bovine serum (FBS) (Euroclone, Pero, Mi, Italy; cat# ECS0180D) (complete medium), seeded and expanded in 25 cm^2^ tissue flasks (Corning, Stone Staffordshire, England, cat# 430639) in a CO_2_ incubator. The adherent cells were evaluated microscopically every 24–48 h and when they reached about 70% confluence were trypsinized, washed and cryopreserved in 90% FBS and 10% dimethyl sulfoxide (DMSO) for later use. To confirm that the isolated cells derived from neoplastic specimens, at least 3 cytospins were carried out exploiting 10^5^ cultured cells/cytospin deriving from 4–6 passages, for morphologic and immunocytochemical characterization, as described in [14, 39].

### Solutions to measure changes in [Ca^2+^]_i_

Physiological salt solution (PSS) consisted of (in mM): 150 NaCl, 6 KCl, 1.5 CaCl_2_, 1 MgCl_2_, 10 Glucose, 10 Hepes. A Ca^2+^-free solution (0Ca^2+^) was obtained by replacing CaCl_2_ with 2 mM NaCl and adding 0.5 mM EGTA. NaOH was used to titrate solutions to pH 7.4. An osmometer (Wescor 5500, Logan, UT) was used to measure the osmolality of the solutions, which was found to be 338 mmol/kg.

### Intracellular Ca^2+^ imaging

TRPA1-mediated changes in [Ca^2+^]_i_ were monitored in mCRC and non-neoplastic cells loaded with the Ca^2+^-sensitive ratiometric indicator, Fura-2 acetoxymethyl ester (Fura-2/AM) [40]. The cells were plated on round glass coverslips (8 mm diameter) coated with collagen (5 mg/mL; Sigma), bathed with PSS, loaded with 4 µM Fura-2 and then maintained in the presence of the Ca^2+^ indicator for 30 min at 37 °C and 5% CO_2_. Subsequently, the cells were extensively washed with fresh PSS and the coverslip was gently attached to the bottom of a Petri dish with silicon grass (Saratoga, Trezzano sul Naviglio, Mi, Italy). The Petri dish was then moved on the stage of an upright epifluorescence Axiolab microscope (Carl Zeiss, Oberkochen, Germany) and the cells were observed with a Zeiss × 40 Achroplan objective (water-immersion, 0.9 numerical aperture, 2.0 mm working distance). Every 3 sec, Fura-2 was alternately (0.5 Hz) excited at 340 and 380 nm, and the emitted fluorescence was recorded at 510 nm. A filter wheel (Lambda 10, Sutter Instrument, Novato, CA, USA) was used to accommodate the excitation filters. 10–40 rectangular “regions of interest” (ROI) were drawn around the cells that were clearly identifiable in the visual field. At each excitation wavelength, images of the visual field and the fluorescence within each ROI were acquired by an Extended-ISIS Camera (Photonic Science, Millham, UK). A custom software that was working in the LINUX environment was employed to control both the Extended-ISIS Camera and the filter wheel. The LINUX-based software was also used to measure the ratio of the mean fluorescence emitted at 510 nm when the cells within each ROI were excited alternatively at 340 and 380 nm (F_340_/F_380_). All recordings were carried out at room temperature (22 °C).

### Mitochondrial Ca^2+^ measurement

Mitochondrial Ca^2+^ was evaluated with Rhod-2/AM by using the same single-cell imaging set-up used to detect variations in Fura-2 fluorescence. Rhod-2 is excited at 545 nm and emits fluorescence at 590 nm. Therefore, changes in Rhod-2 fuorescence were measured by using a TRITC filter cube. The mCRC cells were incubated in PSS containing 4 µM Rhod-2/AM for 45 min at 37 °C and 5% CO_2_. Subsequently, the cells were extensively washed with fresh PSS and the coverslip was attached to the bottom of a Petri dish, as described above for Fura-2. Recordings were performed and plotted on-line every 3 sec. All the recordings were carried out at 22 °C.

### Measurement of mitochondrial membrane potential (ΔΨ_m_)

ΔΨ_m_ was measured as recently described [14], by incubating mCRC cells in PSS containing 25 nM TMRM and 200 nM Cyclosporine H for 30 min at 37 °C and 5% CO_2_. Changes in TMRM fluorescence were recorded by using the same imaging set-up employed to record TRPA1-mediated increases in Fura-2 and Rhod-2 fluorescence. The TMRM red-orange fluorescence (excitation 480 nm, emission 510 nm) was measured with the aid of a TRITC filter cube for live imaging. A round diaphragm was exploited to increase the contrast. Recordings were carried out and plotted on-line every 10 s. The experiments were performed at 22 °C.

### Measurement of caspase-3/7 activity

Intracellular caspase-3/7 activity was evaluated by single-cell fluorescence microscopy by using the CellEvent™Caspase-3/7 Green Detection Reagent according to the manufacturer’s intructions (Thermofisher Scientific, Rodano, Mi, Italy). This reagent consists of a four-amino acid peptide (DEVD) conjugated to a nucleic acid-binding dye, which is non-fluorescent when it is not bound to DNA. The DEVD peptide sequence is a cleavage site for caspase-3/7 and, therefore, upon caspase-3/7 activation in apoptotic cells, the free dye can bind to DNA and emit bright green fluorescence. Cells were seeded in 12-well plates (Corning, Stone Staffordshire, England; cat#3513) and, upon reaching 70% confluence, were loaded with the CellEvent™ Caspase-3/7 Green Detection Reagent (5 µM) for 30 min at 37 °C and 5% CO_2_. After extensive washing, the 12-well plate was moved upon the stage of a Confocal Microscope Leica SP8 equipped with an Okolab stage-top incubator for live cell imaging at 37 °C and 5% CO_2_ and a Leica HC PL Fluotar objective 20x objective (6.9 mm working distance, 0.4 numerical aperture). The experiments were performed at the Confocal Microscopy Facility of the Centro Grandi Strumenti, University of Pavia.

### Immunoblotting

Total protein homogenates from primary mCRC cells were treated with a RIPA buffer containing (150 mM NaCl, 0.5% sodium deoxycholate, 0.1% SDS, 0.1% Triton X-100, 50 mM Tris-HCl, pH 8, and the protease inhibitor cocktail cOmplete (cOmplete Tablets EASYpack, 04693116001; Merck, Milan, Italy). Laemmli buffer was added to the samples, and denaturation was made by heating in a thermal block for 10 min at 80 °C. Twenty µicrograms of proteins were loaded in precast polyacrylamide gel (4–20% Mini-PROTEAN TGX Stain-Free Gels, Bio-Rad, Segrate, Italy) and SDS-PAGE performed [57]. Then, the proteins were transferred out of the gel onto the PVDF Membrane (Trans-Blot Turbo Transfer Pack, #1704156, Bio-Rad, Segrate, Italy) with the Trans-Blot Turbo Transfer apparatus (#1704150, Bio-Rad, Segrate, Italy). Membranes were blocked by incubation for 1 h at 22 °C in Tris-buffered saline with 5% skimmed dry milk and 0.1% Tween (blocking solution). Membranes were incubated overnight with anti-TRPA1 rabbit antibody (PA146159, 1:500 dilution; Thermo Fisher Scientific, Monza, Italy) in the blocking solution. The membranes were washed three times and incubated for 1 h with goat anti-rabbit IgG antibody, peroxidase-conjugated (1:100000; AP132P; Millipore part of Merck S.p.a., Vimodrone, Italy). The detection of the bands was performed with the chemiluminescent substrate kit Westar Supernova Western (CYANAGEN, Bologna, Italy) and the molecular weights of the bands were pinpointed using pre-stained molecular weight markers (#161-0376, Bio-Rad Laboratories, California, USA). Stripped membranes were re-probed by incubating with the housekeeping anti-β-actin rabbit monoclonal antibody (AB-81599, 1: 2000; Immunological Sciences, Rome, Italy) [58]. Protein bands were visualized using the iBright™ CL1000 Imaging System (Thermo Fisher Scientific, Monza, Italy). The band intensity was semi-quantified by using iBright Analysis Software (Thermo Fisher Scientific, Monza, Italy) and the results were expressed as TRPA1 / β-actin ratio.

### Gene silencing

Gene silencing of TRPA1 has been performed by using the same strategy as that employed to down-regulate the expression of STIM1 and Orai1 [40], TRP Vanilloid 1 [14] and two-pore channel 1 [13] in primary cultures of mCRC cells. The esiRNA targeting TRPA1 was purchased from Sigma-Aldrich Inc. MISSION®esiRNA (human TRPA1) (EHU040601). Negative controls were made by using scrambled siRNA. In brief, when mCRC cells reached 50% confluency, the medium was replaced with reduced serum medium Opti-MEM, (Life Technologies, Milan, Italy). The solution of siRNAs diluted (100 nM final concentration) with Opti-MEM was combined with Opti-MEM containing the Lipofectamine™ transfection reagent (Life Technologies, Milan, Italy), following the manufacturer’s instructions. This solution containing siRNA was incubated for 20 min at room temperature. Finally, the siRNA-Lipofectamin complex was added to the cells and the cells were then left in a CO_2_ incubator for 5 h. The siRNA-Lipofectamin complex was then eliminated, and fresh culture media was added to the cells. Protein silencing was effective 48 h after transfection. To check the knockdown efficiency, immunoblot for TRPA1 was performed in siRNA and Mock treated cells (see Figs. S3 and S4).

### Statistics

All the data have been generated by mCRC and non-neoplastic cells expanded from three distinct patients. Each experiment has been carried out three times by using cells obtained by each patient in three separate days. The amplitude of cytosolic and mitochondrial Ca^2+^ signals evoked by each agonist (AITC, H_2_O_2_, and 4-HNE) was measured as the difference between the F_340_/F_380_ ratio at the peak of the Ca^2+^ signal and the mean *F*_340_/*F*_380_ ratio of 1 min baseline recorded before addition of the agonist. The dose-response relationship reported in Fig. S5B was fitted by using the equation [59]:

1 $$Y = \frac{{100}}{{1 + \frac{{{\mathrm{EC}}_{50}}}{{\left[ {{{{\mathrm{H}}}}202} \right]}}}}$$

where *Y* is the amplitude of the Ca^2+^ response, [H_2_O_2_] is the H_2_O_2_ concentration, and EC_50_ is the half-maximal effective concentration.

Pooled data are presented as mean ± SE. The number of cells analyzed for each condition is indicated in the corresponding bar histograms. Normality of the data was tested with Shapiro–Wilk test. If the data distribution was normal, differences between two groups were evaluated by using the Student’s *t*-test for unpaired observations, whereas Differences between multiple groups were evaluated by using one-way ANOVA analysis followed by the post hoc Dunnett’s or Bonferroni tests as appropriate. *p* < 0.05 indicated statistical significance. No statistical methods were used to predetermine the sample size.

### Chemicals

Fura-2/AM and Rhod-2/AM were purchased from Invitrogen (Life Technologies). All the chemicals were purchased from Sigma-Aldrich .

## Supplementary information

Supplemental Material

Original Data File_Figure 1A

Original Data File_Figure 1A_Beta-actin

Original Data File_Figure S3A_TRPA1 silencing

Original Data File_Figure S3A_Beta-actin silencing

## Data Availability

All the data generated or analyzed in this study are available upon reasonable request to the corresponding authors.
