# Supplementary material for: Transient receptor potential ankyrin 1 (TRPA1) mediates reactive oxygen species-induced Ca2+ entry, mitochondrial dysfunction, and caspase-3/7 activation in primary cultures of metastatic colorectal carcinoma cells
Source: Cell Death Discov. 2023 Jul 1;9:213. doi: 10.1038/s41420-023-01530-x (PMC10314907; doi:10.1038/s41420-023-01530-x)
Supplement: Supplementary file 1 — Supplemental Material [file 41420_2023_1530_MOESM1_ESM.docx]

**Supplemental material**


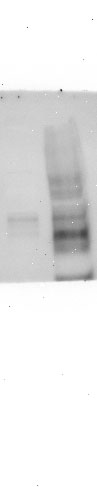


**Figure S1A. Full-length uncropped Western blot corresponding to Figure 1A (TRPA1 protein).**

**
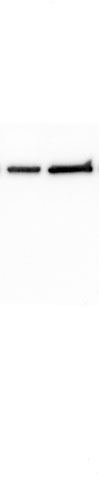
**

**Figure S1B. Full-length uncropped Western blot corresponding to Figure 1A (β-actin protein).**

**Figure S2. AITC evokes extracellular Ca^2+^ entry in primary cultures of mCRC cells.** AITC (30 µM) failed to elicit intracellular Ca^2+^ signaling when the mCRC cells were bathed in the absence of extracellular Ca^2+^ (0Ca^2+^), whereas the Ca^2+^ response arose upon restitution of extracellular Ca^2+^ to the perfusate.

**
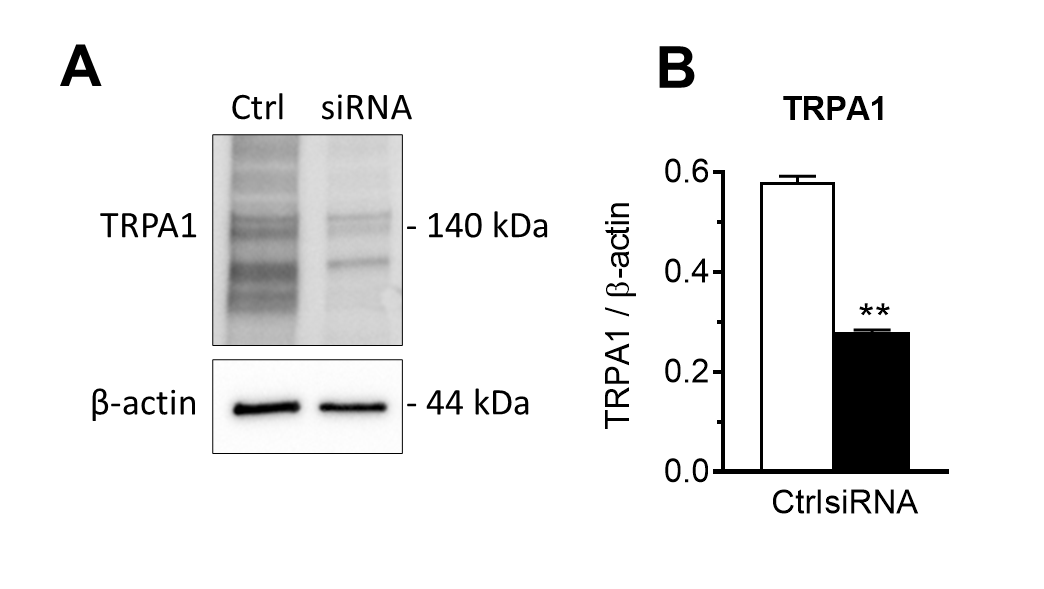
**

**Figure S3. *TRPA1* gene silencing via a selective esiTRPA1 reduces the expression of TRPA1 protein in primary cultures of mCRC cells**. (A) mCRC cells were transfected with short interfering RNA (siRNA) selectively targeting TRPA1 expression and a scrambled siRNA (Ctr), as described in “Materials and methods”. Blots representative of four were shown. Lanes were loaded with 20 μg of proteins, probed with affinity‐purified antibodies and processed as described in “Materials and methods”. The same blots were stripped and reprobed with anti‐β‐actin (β-actin) polyclonal antibody, as housekeeping. Major bands of the expected molecular weights were indicated. (B) Densitometric analysis of the bands revealed a significant reduced TRPA1 protein expression in silenced cells compared to controls (52%, reduction; Student’s *t*-test: ** *p* < 0.01; Student’s t test). Densitometry was performed by Total Lab V 1.11 computer program (Biosciences Europe, Little Chalfont, UK), and the results were normalized to the corresponding β-actin.

**
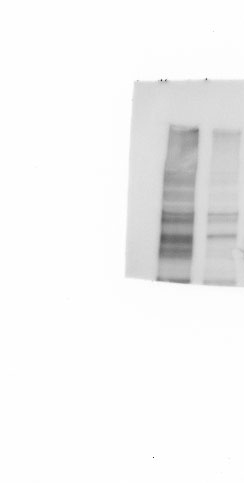
**

**Figure S4A. Full-length uncropped Western blot corresponding to Figure S3A (silencing of TRPA1 protein).**

**
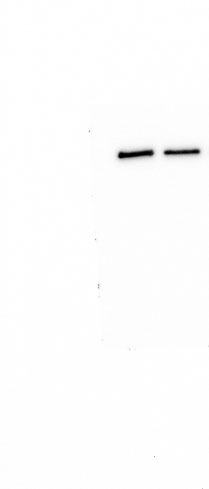
**

**Figure S4B. Full-length uncropped Western blot corresponding to Figure S3A (silencing of β-actin protein).**


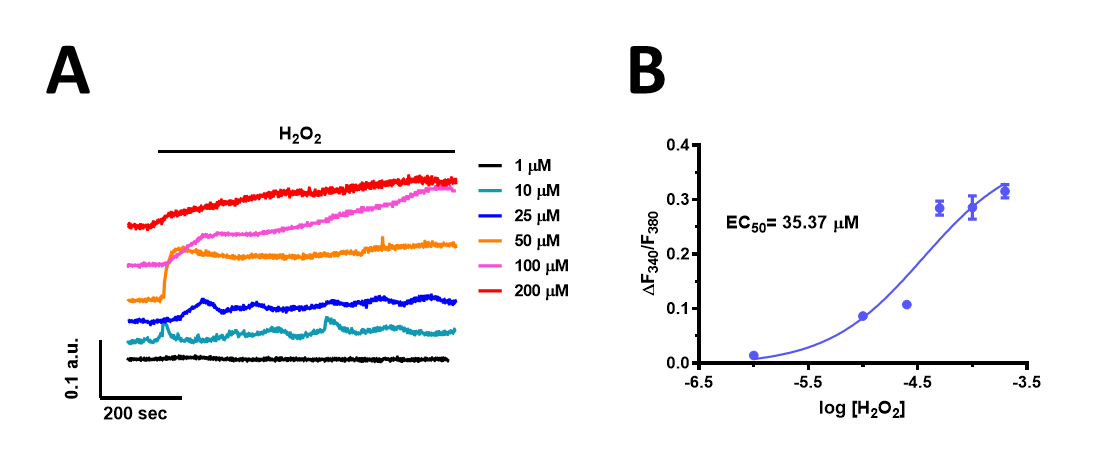


**Figure S5. Dose-response relationship of H_2_O_2_-evoked intracellular Ca^2+^ signals in primary cultures of mCRC cells.** (A) Intracellular Ca^2+^ signals induced by increasing concentrations of H_2_O_2_ in primary cultures of mCRC cells. The baseline of Ca^2+^ tracings has been shifted to avoid their overlapping for representation purposes. (B) Dose–response relationship of the amplitude of H_2_O_2_-evoked Ca^2+^ signals in mCRC cells. obtained from a fit to a sigmoidal concentration‐response curve by using Equation (1). The number of cells analyzed for each concentration ranged from 39 to 285 from three independent experiments.

**
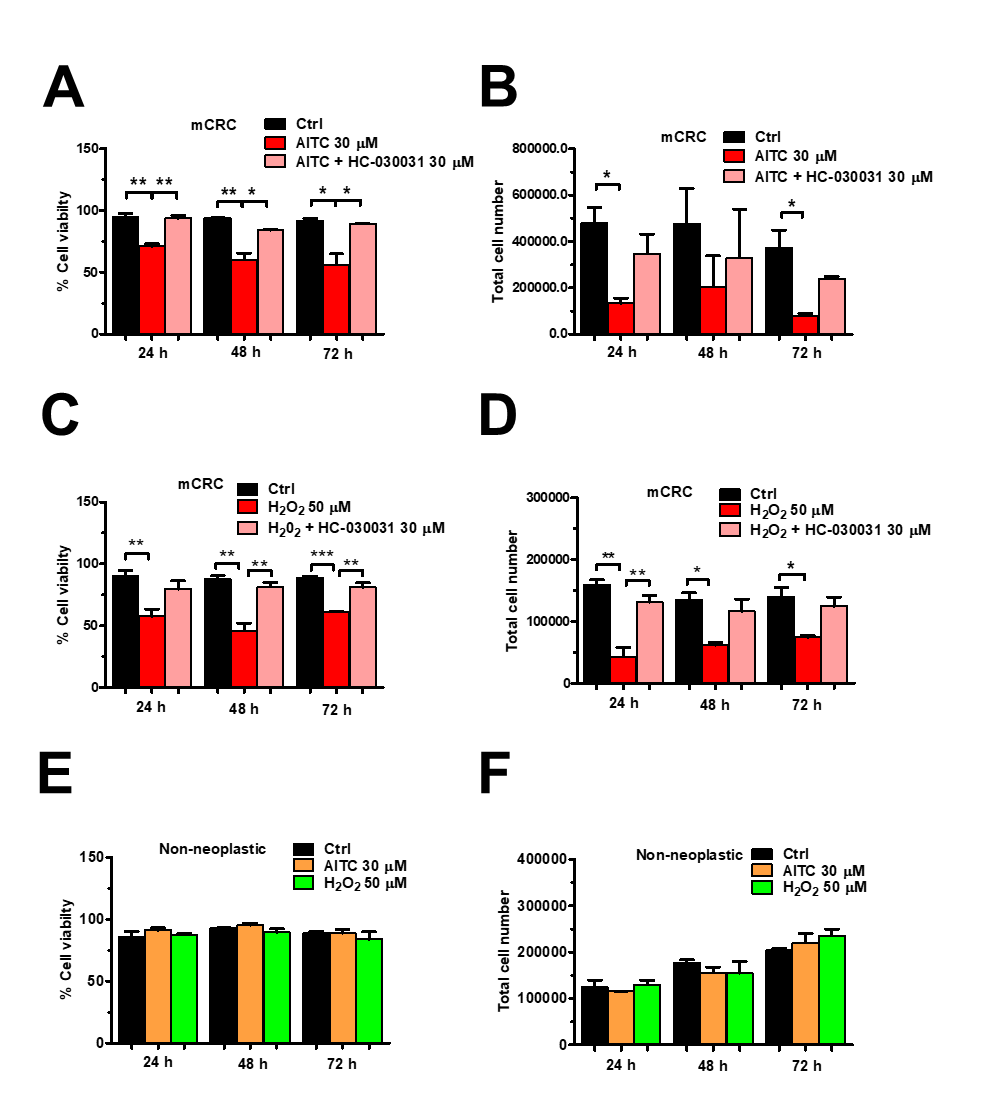
**

**Figure S6. TRPA1 mediates H_2_O_2_-induced reduced in cell viability and proliferation in mCRC but not non-neoplastic cells.** (A) Mean±SE of the percentage of viable mCRC cells upon 24 h, 48 h, and 72 h exposure to AITC (30 µM) in the presence and absence of HC-030031 (30 µM). Control (Ctrl) cells were not exposed to either AITC or HC-03031. One-way ANOVA analysis followed by the post-hoc Bonferroni. ** *p* < 0.01 and * *p* < 0.05. N=4 for each experimental condition. (B) Mean±SE of the total number of mCRC cells upon 24 h, 48 h, and 72 h exposure to AITC (30 µM) in the presence and absence of HC-030031 (30 µM). Control (Ctrl) cells were not exposed to either AITC or HC-03031. One-way ANOVA analysis followed by the post-hoc Bonferroni. * *p* < 0.05. N=4 for each experimental condition. (C) (A) Mean±SE of the percentage of viable mCRC cells upon 24 h, 48 h, and 72 h exposure to H_2_O_2_ (50 µM) in the presence and absence of HC-030031 (30 µM). Control (Ctrl) cells were not exposed to either H_2_O_2_ or HC-03031. One-way ANOVA analysis followed by the post-hoc Bonferroni. *** *p* < 0.001 and ** *p* < 0.01. N=4 for each experimental condition. (D) Mean±SE of the total number of mCRC cells upon 24 h, 48 h, and 72 h exposure to H_2_O_2_ (50 µM) in the presence and absence of HC-030031 (30 µM). Control (Ctrl) cells were not exposed to either H_2_O_2_ or HC-03031. One-way ANOVA analysis followed by the post-hoc Bonferroni. ** *p* < 0.01 and * *p* < 0.05. N=4 for each experimental condition. (E) Mean±SE of the percentage of viable non-neoplastic cells upon 24 h, 48 h, and 72 h exposure to AITC (30 µM) or H_2_O_2_ (50 µM). Control (Ctrl) cells were not exposed to either AITC or H_2_O_2_. (F) Mean±SE of the total number of non-neoplastic cells upon 24 h, 48 h, and 72 h exposure to AITC (30 µM) or H_2_O_2_ (50 µM). Control (Ctrl) cells were not exposed to either AITC or H_2_O_2_.

**
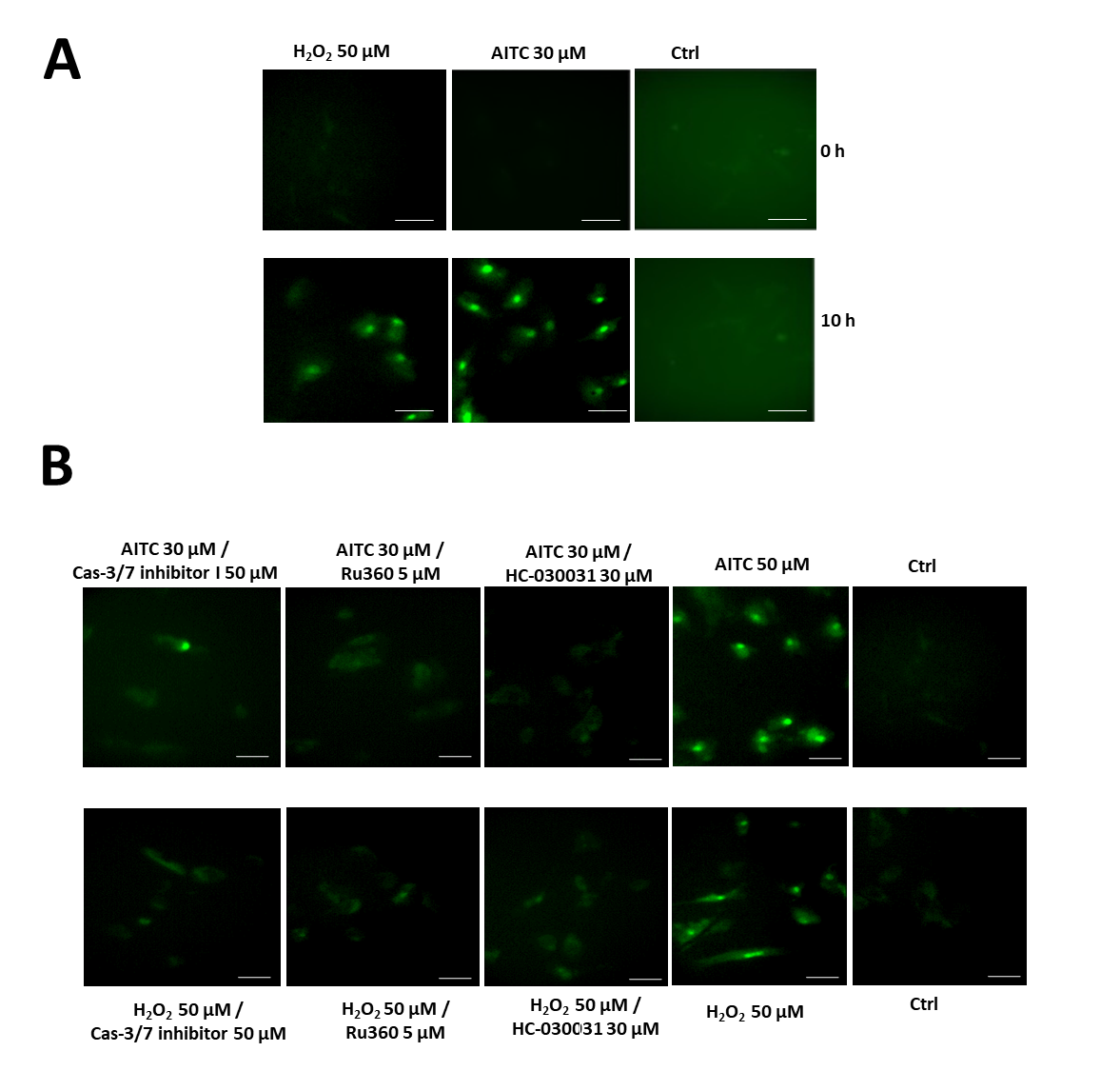
**

**Figure S7. TRPA1-mediated Ca^2+^ signals induce caspase-3/7 activation in primary cultures of mCRC cells.** (A) Representative images reporting caspase-3 activation in mCRC cells loaded with the CellEvent^TM^ fluorescent dye and stimulated with either AITC (30 µM) or H_2_O_2_ (50 µM). Caspase-3 activity was evaluated before (0 h) and at 10 h after TRPA1 activation. Scale bars, 60 µm. (B) Representative images reporting caspase-3/7 activation in mCRC cells loaded with the CellEvent^TM^ fluorescent dye and stimulated with either AITC (30 µM; upper panel) or H_2_O_2_ (50 µM; lower panel) in the presence of any of the following drugs: HC-030031 (30 µM); Ru360 (5 µM), and Caspase-3/7 Inhibitor I (50 µM). Scale bars, 60 µm.
