# Supplementary material for: Transient receptor potential ankyrin 1 (TRPA1) mediates reactive oxygen species-induced Ca2+ entry, mitochondrial dysfunction, and caspase-3/7 activation in primary cultures of metastatic colorectal carcinoma cells
Source: Cell Death Discov. 2023 Jul 1;9:213. doi: 10.1038/s41420-023-01530-x (PMC10314907; doi:10.1038/s41420-023-01530-x)
Supplement: Supplementary file 2 — Original Data File_Figure 1A [file 41420_2023_1530_MOESM2_ESM.docx]

**Supplemental material**


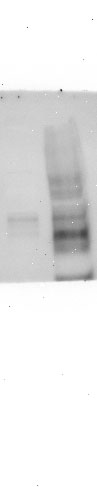


**Figure S1A. Full-length uncropped Western blot corresponding to Figure 1A (TRPA1 protein).**
