# Supplementary material for: Transient receptor potential ankyrin 1 (TRPA1) mediates reactive oxygen species-induced Ca2+ entry, mitochondrial dysfunction, and caspase-3/7 activation in primary cultures of metastatic colorectal carcinoma cells
Source: Cell Death Discov. 2023 Jul 1;9:213. doi: 10.1038/s41420-023-01530-x (PMC10314907; doi:10.1038/s41420-023-01530-x)
Supplement: Supplementary file 4 — Original Data File_Figure S3A_TRPA1 silencing [file 41420_2023_1530_MOESM4_ESM.docx]

**Supplementary material**

**
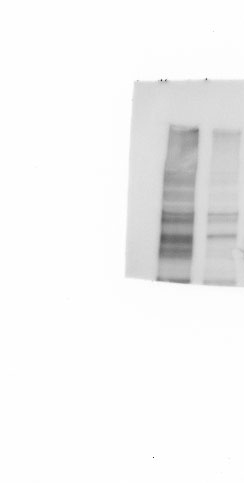
**

**Figure S4A. Full-length uncropped Western blot corresponding to Figure S3A (silencing of TRPA1 protein).**
